# The Extracellular Domain of Pollen Receptor Kinase 3 is structurally similar to the SERK family of co-receptors

**DOI:** 10.1038/s41598-018-21218-y

**Published:** 2018-02-12

**Authors:** Sayan Chakraborty, Haiyun Pan, Qingyu Tang, Colin Woolard, Guozhou Xu

**Affiliations:** Department of Molecular and Structural Biochemistry, North Carolina State University, 128 Polk Hall, Raleigh, 27695 USA

## Abstract

During reproduction in flowering plants, the male gametophyte delivers an immotile male gamete to the female gametophyte in the pistil by formation of pollen tubes. In *Arabidopsis thaliana*, two synergid cells situated on either side of the egg cell produce cysteine-rich chemoattractant peptide LURE that guides the pollen tube to the female gametophyte for sexual reproduction. Recently, in *Arabidopsis thaliana*, Pollen Receptor Kinase 3 (PRK3), along with PRK1, PRK6, and PRK8, have been predicted to be the receptors responsible for sensing LURE. These receptors belong to the Leucine Rich Repeat Receptor Like Kinases (LRR-RLKs), the largest family of receptor kinases found in *Arabidopsis thaliana*. How PRKs regulate the growth and development of the pollen tube remains elusive. In order to better understand the PRK-mediated signaling mechanism in pollen tube growth and guidance, we have determined the crystal structure of the extracellular domain (ecd) of PRK3 at 2.5 Å, which resembles the SERK family of plant co-receptors. The structure of ecdPRK3 is composed of a conserved surface that coincides with the conserved receptor-binding surface of the SERK family of co-receptors. Our structural analyses of PRK3 have provided a template for future functional studies of the PRK family of LRR-RLK receptors in the regulation of pollen tube development.

## Introduction

Sexual reproduction in plants is an intricate process involving pollen-pistil interactions between male and female gametophytes. Through pollen tube formation, the male gametophyte is directed towards the female gametophyte in the pistil^1^. The pollen tube is a tip growing cell that originates from the pollen grain^2^. The pollen tube then penetrates the surface of the stigma and the style; it reaches the ovule for fertilization and delivers immotile male gametes^1,2^. One sperm cell fertilizes the egg cell, while another sperm cell fuses with the central cell to produce the embryo and endosperm^3^. This highly complex process is thought be regulated by several external and internal cues^4^. The signaling cascade controlling the pollen tube growth ensures successful fertilization by providing directionality and guidance. It not only protects polyspermy, but also stops the arrival of an incompatible pollen tube^2^. Lipid-transfer protein, Transmitting Tissue-Specific protein (TTS), and Styler Pectin all have been implicated as crucial factors in this signaling process^5–8^. After the identification of the first pollen specific receptor-like kinase from *Petunia inflata*, more receptor like kinases such as LePRK1 and LePRK2 were isolated from *Lycospersicon esculentum*^9,10^. The study of LePRK1 and LePRK2 proved that these two receptor like kinases play crucial roles in the pollen tube growth, pollen-pistil interaction, and pollen tube localization^10^. This clearly indicated that receptor like kinases are strongly involved in the pollen-pistil interaction and plant sexual reproduction.

Plant receptor-like kinases (RLKs) play key roles in various biological processes involving plant immune defense and development. This includes defense against pathogens, stomata formation, organ and flower development, xylem development, etc.^11–15^. Generally, the RLK proteins have an extracellular domain, a transmembrane domain, and a kinase domain with Ser/Thr-type specificity^16,17^. Both LePRK1, LePRK2 and pollen specific receptor kinases from *Petunia inflata* belong to the family of Leucine-Rich-Repeat Receptor-Like Kinases (LRR-RLKs)^9,10,18^. Bioinformatics studies on Arabidopsis genome, conducted by McCormick *et al*., presented multiple pollen receptor kinase candidates that have similar amino acid sequences and topologies when compared to the LePRKs. The LRR-RLK subfamily is the largest group of RLKs in Arabidopsis, and they are involved in mediating protein-protein and protein-ligand interactions^19–22^.

The presence of numerous receptor kinases in the pollen tube has long puzzled biologists. It was hypothesized that a chemical or biological factor secreted from the ovule is perceived by the RLKs and that in turn regulates the pollen tube guidance^23^. Various small molecules and modified peptides were thought to be involved in these cell-cell communications and signal transduction^24–26^. LAT52 and LeSTIG1 of *Solanum lycopersicum* as well as SCR/SP11 of *Brassica sp* were thought to be involved in pollen tube germination and growth^27–32^. LAT52 an essential peptide required for pollen hydration and pollen tube formation was found to be interacting with the extracellular LRR domain of LePRK2^29^. McCormick *et al*. demonstrated that LAT52 interacts with LePRK2. This was a major step in understanding the role of pollen receptor kinases in pollen tube growth as well as pollen tube guidance and overall plant reproduction^28,29^. Not long ago it was discovered that two synergid cells situated on either side of the egg cell of *Torenia fournieri* produce cysteine rich chemoattractant peptides LUREs that guide the pollen tube to the female gametophyte for sexual reproduction^33–35^. The predicted mature peptides of LURE1 and LURE2 have six cysteine residues; the correct intramolecular disulfide bond formation is essential for their activity. There is a significant amino acid divergence between LURE1 and LURE2 except for the conserved cysteines. As LURE 1 and LURE 2 are highly diverged, it is suggested that they may bind to different receptors^34^. Like *Torenia fournieri*, defensin like LURE peptides are also found in Arabidopsis which are named AtLURE1^35,36^. Interestingly, six genes including a pseudo gene encoding AtLURE1 peptides (AtLURE1.1–1.5) form a species-specific gene cluster in the genome. Four of the five AtLURE1 peptides have been demonstrated to possess pollen tube attractant activity, although AtLURE1.1 has a relatively lower activity. On the other hand, AtLURE1.5 peptide which lacks one of the six conserved cysteine residues does not have pollen tube guaidance activity^36^.

In *Arabidopsis thaliana*, AtPRK3, along with AtPRK1, AtPRK6, and AtPRK8, have been predicted as the receptors that sense the AtLURE1.2 peptide. It has been revealed that the AtPRK3 has a vital role in pollen tube growth, plant fertility, and plant reproduction^37^. In addition, Male Discoverer1 (MDIS1), MDIS1-Interacting Receptor-Like Kinase1 (MIK1), and MIK2, have been independently identified by another research group as potential LURE1 receptors in Arabidopsis^38^.

While a lot of valuable data about peptide ligands and LRR-RKs have been obtained from genetic and biochemical experiments, visualization of ligand/LRR-RK complex structures at the atomic level is vital to understand the functions of LRR-RKs and their mediated biological processes^39^. Here we present the atomic structure of the extracellular domain (ecd) of the PRK3 (residues 20–237) from *Arabidopsis thaliana* resolved at 2.5 Å. Structural elucidation of the ecdAtPRK3 will provide insight into its function, aid in identifying ligand or receptor binding site, and will describe the role of other PRKs in the regulation of pollen tube development and function.

## Results

### Structure of the extracellular domain of PRK3

**Figure 1:**
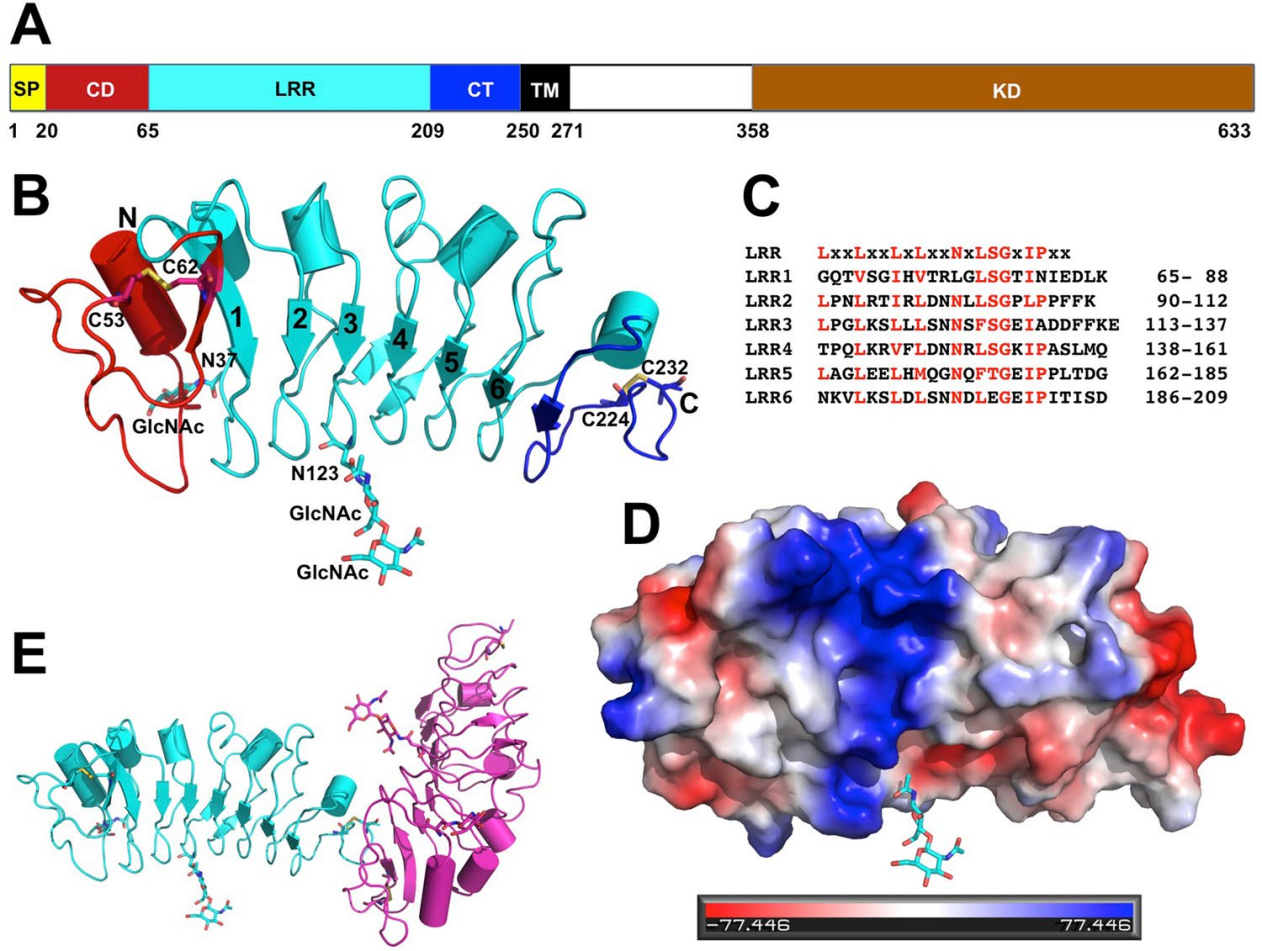
Structure of the extracellular domain of *Arabidopsis thaliana* PRK3. (**A**) PRK3 contains an N-terminal signal peptide domain (SP, residues 1–19), an LRR capping domain (CD, 20–54), a leucine rich repeat domain (LRR, 65–209) which harbors 6 plant-specific LRRs, an LRR C-terminal domain (CT, 210–249), a transmembrane domain (TM, 250–270), and an intracellular kinase domain (KD, 358–633). (**B**) The structure of PRK3 (residues 20–237) is represented as a cartoon representation. The CD is colored in red, LRR domain in cyan, and CT in blue. Each LRR is numbered in the N-terminal β-strand of the repeat. Two glycosylated asparagine residues and the four-cysteine residues that form two disulfide bonds of PRK3 are depicted as stick representations and the disulfide bonds are colored in yellow. (**C**) Sequence alignment of the six LRRs in the ectodomain of PRK3; the conserved residues are colored in red. The conserved LRR motif is shown on the top of the alignment where “X” stands for any residue. (**D**) Surface representation of the PRK3 structure where the positively charged surfaces are depicted in blue, and negatively charged surfaces are colored in red. (**E**) The asymmetric unit of the PRK3 crystal contains two chains of the PRK3 molecule, with the A chain colored in cyan and B chain shown in red.

PRK3 is a type I transmembrane receptor which contains an N-terminal signal peptide domain (SP), an LRR capping domain (CD), a leucine rich repeat domain (LRR), an LRR C-terminal domain (CT), a transmembrane domain (TM), and an intracellular kinase domain (KD) (Fig. 1A). To gain a better understanding of PRK3-mediated pollen tube development, we have crystallized a protein fragment of PRK3 spanning residues 20–237 in space group P4_2_ with two PRK3 protein molecules in each asymmetric unit (Table 1 and Fig. 1). The AtPRK3 ectodomain contains capped regions shielding the hydrophobic patches from solvent accessibility at the N terminal and C terminal regions; these regions are termed the LRR capping domain and LRR C-terminal domain, respectively^40^. The capped regions are also suggested to maintain structural integrity. There are two pairs of cysteine residues, C53 and C62, C224 and C232; they form two disulfide bonds in the CD and the CT, respectively^40–43^. The C53–C62 disulfide bond stabilizes the conformation of the CT. A similar disulfide bond, C57–C64, is present in the LRR capping domain of Brassinosteroid Insensitive 1-Associated Receptor Kinase 1 (BAK1). The AtPRK3 ectodomain disulfide bonding pattern is similar to that of Somatic Embryogenesis Receptor Kinase 1 (SERK1) and SERK2. SERK1 has two disulfide bonds: one in the LRR capping domain between C58–C65 and C202–C210 located in the LRR C terminal domain. In the case of SERK2, C61–C68 and C205–C213 form disulfide bonds in the LRR capping domain and in the LRR C-terminal domain respectively. Mutations in the cysteine residues have been shown to affect the functions of FLS2, whereas the same mutations in CLV2 do not impair its function^44–46^. It has been predicted that these cysteine residues take part in protein folding, trafficking, and the binding of other proteins^47,48^.

**Table 1:** Data collection and refinement statistics.

**Data collection**	AtPRK3 extracellular domain
	Native
Beam line	APS 22-ID
Wavelength (Å)	1.0000
Space group	P4_2_
Cell dimensions a, b, c (Å), α, β, γ (°)	72.061 72.061 125.677, 90 90 90
Resolution (Å)	50–2.496 (2.585–2.496)
Total reflections	167703 (15824)
Unique reflections	22282 (2214)
Redundancy	7.5 (7.1)
Completeness (%)	99.83 (99.86)
I/σ	24.52 (4.75)
CC1/2	0.999 (0.943)
CC*	1 (0.985)
R-meas (%)	0.07334 (0.4684)
R-pim (%)	0.02655 (0.1723)
R-merge (%)	0.06831 (0.4351)
**Refinement**	
Resolution range (Å)	39.578–2.496 (2.585–2.496)
Reflections used in refinement	22267 (2214)
Reflections used in R-free	1089 (113)
Number of non-hydrogen atoms	3357
R(work)	0.2380 (0.2536)
R(free)	0.2738 (0.3319)
CC(work)	0.922 (0.840)
CC(free)	0.902 (0.684)
Mean B-factor (Å^2^), overall	53.56
Bonds (Å)	0.008
Angles (°)	1.18
Ramachandran Favored (%)	92.48
Ramachandran Allowed (%)	7.04
Ramachandran Outliers (%)	0.49

Highest resolution shell is shown in parenthesis.

It is known that the conserved amino acid residues within the LRR provide a structural backbone, while the non-conserved residues provide variability in the functional repertoire^19,49^. Based on the conserved and non-conserved residues present in the LRR domain, LRRs have been classified into several families^19^. The AtPRK3 ectodomain belongs to the plant specific LRR family^19^. The AtPRK3 ectodomain contains six copies of plant specific LRR repeats ranging from 23–25 amino acid residues (Fig. 1B,C)^19,40^. The conserved LRR sequence for AtPRK3 is LxxLxxLxLxxNxLSGxIPxx. The ectodomain forms a single continuous structure in an arc shaped conformation. The inner face of the arc forms a concave surface, the majority of which contain an extended parallel β sheet. The outer face forms a convex side mostly consisting of various secondary structures such as α-helices, loops and turns^40^.

Two N-glycosylation sites are identified on residues N37 and N123, with only one visible GlcNAc sugar residue conjugated on N37 and two such residues on N123 (Fig. 1B). N-linked glycosylation is quite common in the ectodomains of plant LRRs^50^. Indeed, several N-glycosylation sites have been detected in the LRR ectodomains of FLS2, EFR, and BAK1 receptors that recognize microbe associated molecular patterns (MAMPs)^51,52^. N-glycosylation associated with the polypeptides in the endoplasmic reticulum ensures proper protein folding^53^. In the case of Arabidopsis, abnormal and altered N-glycosylations can affect the abiotic stress response and hamper proper plant development^54–56^. Conserved N-glycosylation patterns, especially NX(S/T) motifs, have been suggested to be important for correct ectodomain structure and function^57^. In the PRK3 ectodomain, the N37 glycosylation, which is present in the α-helical part of the LRR convex side, belongs to the conserved NX(S/T) motif and is believed to be essential for its correct structure and function. However, the N123 glycosylation also belongs to the conserved NX(S/T) glycosylation motif, but is present at the bottom of the concave surface. We believe that the exposed N123 glycosylation pattern on the concave side of the PRK3 ectodomain can form hydrated branches and are likely to facilitate the association with other molecules^58^. Further studies elucidating PRK3 glycosylation patterns are required to comprehend their actual function.

There is a positively charged electrostatic patch on the concave surface of the LRR domain, whereas the C terminal region is mostly negatively charged (Fig. 1D). The positively charged patch on the concave side is likely due to the presence of an abundant number of lysine and arginine residues at positions K64, R75, K88, R94, R97, K112, K117, R142, R143, and R150, which are conserved in most of the PRK3 orthologs from *Arabidopsis thalian* to *Cajanus cajan*. From the previously published LRR ectodomain structures, it has been demonstrated that the concave surface residues generally interact with other proteins or ligands^50^. Therefore, we can safely suggest that the positively charged surface will provide a favorable interacting surface for negatively charged proteins.

### The extracellular domain of PRK3 is monomeric in the crystal

**Figure 2:**
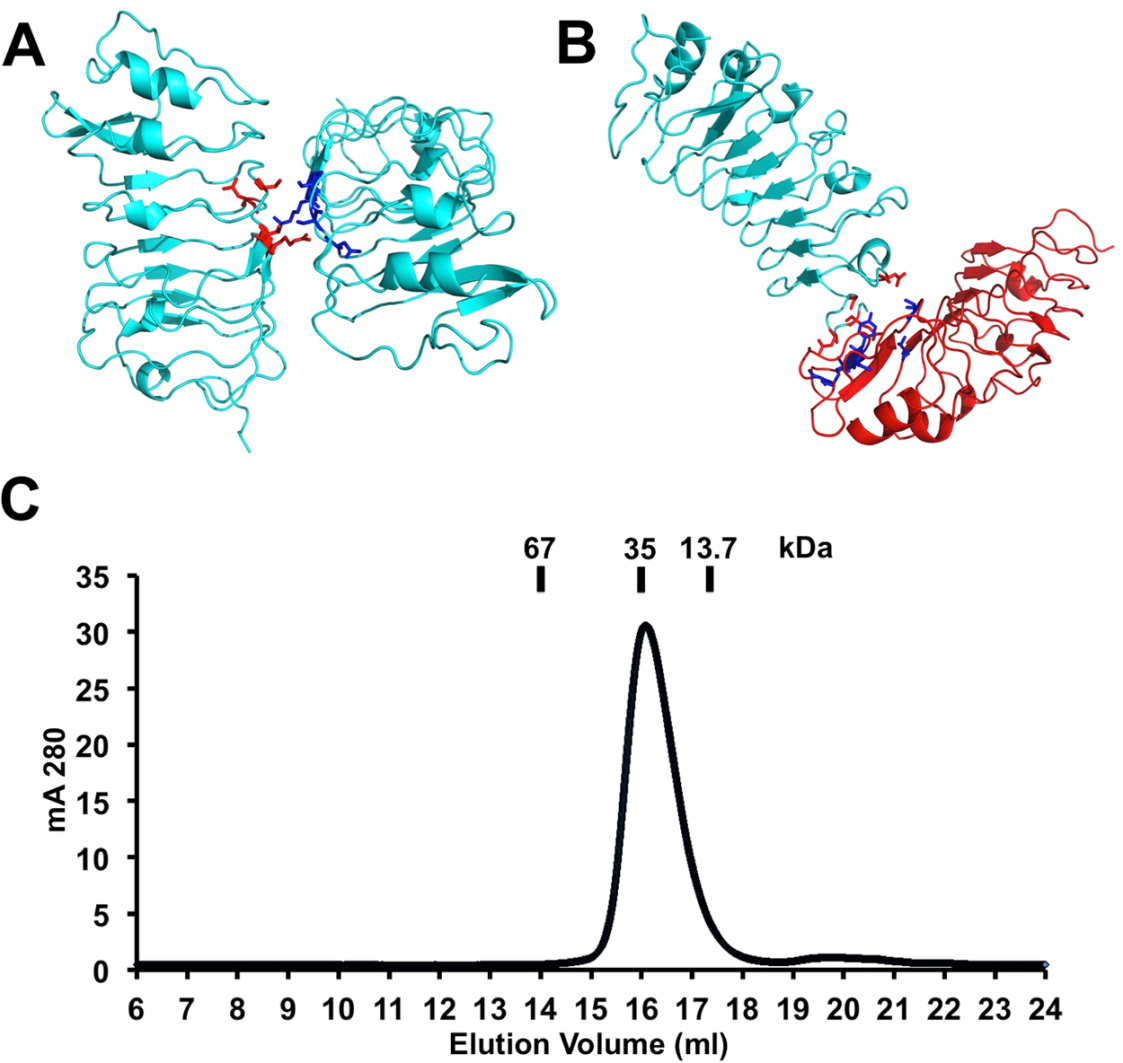
Two crystal packing dimers of the PRK3 structure. (**A**) A crystallographic packing dimer between the same chains (chain A) is depicted in cyan as a cartoon representation, and the residues facilitating the packing interactions are shown in either red or blue with side chains depicted as sticks. (**B**) Cartoon representation of the crystallographic packing dimer between the two different chains (chain A and B), where chain A is depicted in cyan and chain B is in red. The residues mediating the packing interactions are shown in either red or blue with the side chains depicted as sticks. (**C**) Size-exclusion chromatogram of the expressed ecdPRK3 recombinant protein. The retention volumes of proteins used as molecular weight standards are indicated on top.

There are two copies of the PRK3 molecules in the asymmetric unit of the crystal, which are termed chain A and B (Fig. 1E). When we carefully examined the packing interfaces between the chains, we identified two major crystallographic packing dimers (Fig. 2). The largest buried packing interfaces between protein molecules are 494 Å^2^ (between the same chains A/A or B/B, Fig. 2A) and 474 Å^2^ (between A and B chains, Fig. 2B), each represents only about 5% of the total protein surface. The surface residues on packing interfaces are mediated mostly by weak van der Waals interactions. Coupling this fact along with the knowledge of the small area of the binding interface indicates that neither dimers are not stable enough in solution to form dimers or higher order oligomers. Our size-exclusion chromatographic analysis of the recombinant PRK3 protein further supports the hypothesis that ecdPRK3 remains a monomer in solution (Fig. 2C). Based on previous crystallographic studies of the extracellular domains of other plant LRR-RLKs, most of them form monomers in the crystals, which is consistent with our observation of the PRK3 structure^50,59–62^.

### Structural comparison of ectodomains between the SERK family and AtPRK3

The extracellular domain of PRK3 is similar in size to the Somatic Embryogenesis Receptor Kinase (SERK) family of plant LRR-RLKs. The SERK family of proteins are involved in the regulation of immune responses in plants^62,63^. SERK proteins play a significant role in triggering the immune response through the interaction with Pattern Recognition Receptors like FLS2. Moreover, SERK proteins have also been found to be required in order to mount a response to damage associated molecular patterns^62,63^. It is also important to keep in mind that BAK1, SERK1, and SERK2 interact with numerous LRR-RLKs and control multiple signaling networks in the plant body, which illustrates the value of all three proteins within the organism as a whole^62,64,65^.

**Figure 3:**
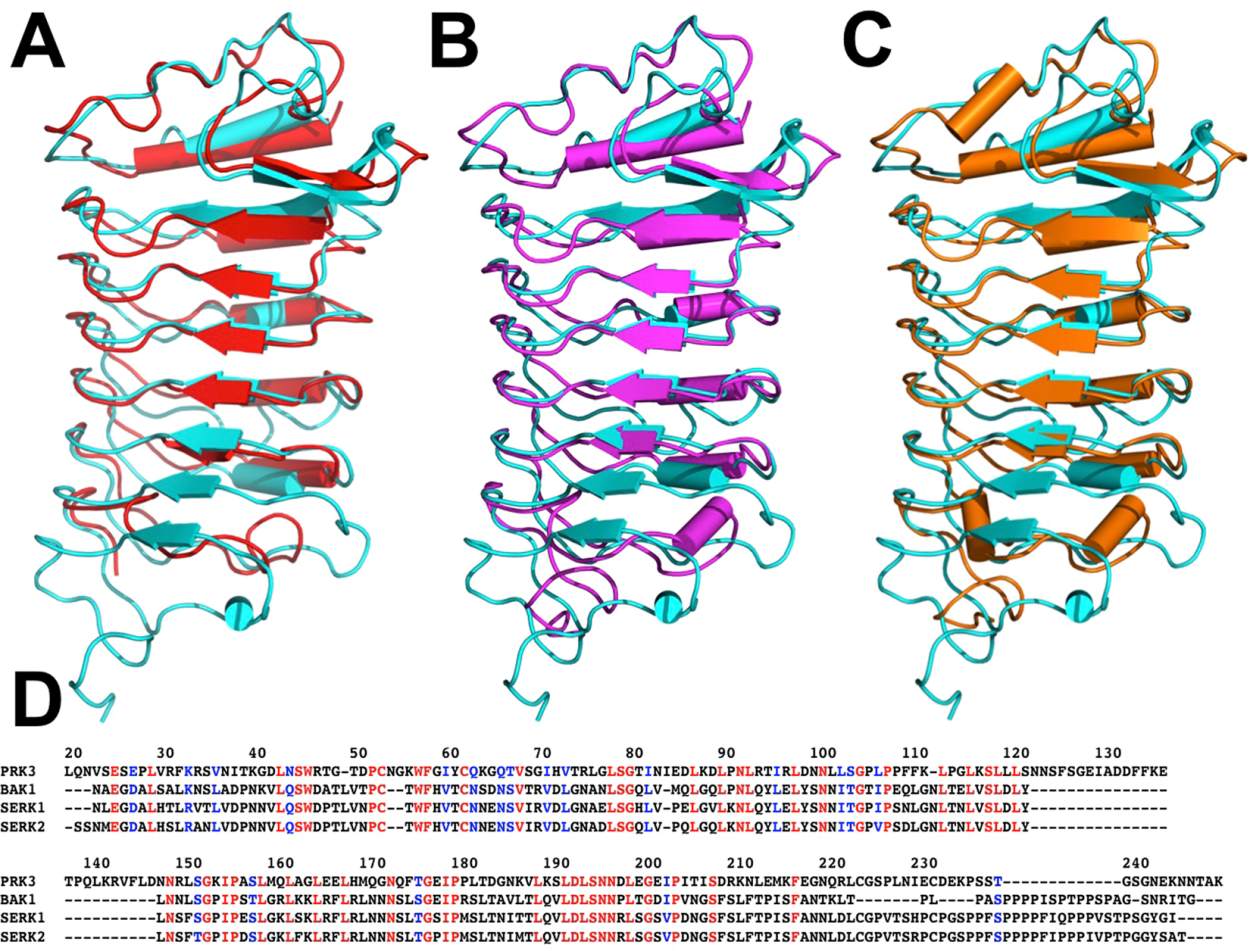
Structural alignment between AtPRK3 and the SERK family of plant co-receptor structures. (**A**) Structure of AtPRK3 is superimposed on BAK1 (PDB ID: 4M7E) with a root-mean-square deviation (RMSD) of 1.92 Å in 154 over 208 residues. The PRK3 structure is colored in cyan and the BAK1 structure is in red. (**B**) Structure of PRK3 is superimposed on SERK1 (PDB ID: 4LSX) with an RMSD of 1.89 Å in 157 over 208 residues. The PRK3 structure is colored in cyan and the SERK1 structure is in magenta. (**C**) Superposition of the ectodomain structures of PRK3 and SERK2 (PDB ID: 5GQR) with an RMSD of 1.96 Å in 159 over 208 residues. The PRK3 structure is colored in cyan and the SERK2 structure is in orange. (**D**) Amino acid sequence of the *Arabidopsis thaliana* PRK3 extracellular domain is aligned with the SERK family of *Arabidopsis thaliana* co-receptors, BAK1, SERK1, and SERK2. Residue numbers of *A. thaliana* PRK3 are indicated on the top of the sequences. The residues that are identical in all four sequences are colored in red. The residues that are similar in all four sequences are colored in blue. Similar residues are defined as: (1) negatively charged side chains as D and E; (2) positively charged side chains as R and K; (3) aliphatic side chains as L, I, and V; (4) aromatic side chains as F, Y, and W; (5) side chains with hydroxyl group as S and T; (6) amide side chains as Q and N. The definition of similar residues is adapted from the BLOSUM matrix^82^. The sequence identity between ecdPRK3 and ecdBAK1, ecdSERK1, and ecdSERK2 are 32.43%, 32.32%, and 30.81% respectively.

The crystal structures of the ectodomain of three Arabidopsis SERK proteins, SERK1, SERK2 and SERK3/BAK1, have previously been determined^59,65,66^. Arabidopsis BAK1, SERK1, and SERK2 have an extracellular domain of 213, 216 and 220 residues, respectively. The extracellular domains of BAK1, SERK1, and SERK2 also share substantial sequence similarity with the PRK3 extracellular domain. The amino acid sequence identity between ecdPRK3 and ecdBAK1, ecdSERK1, and ecdSERK2 is 32.43%, 32.32%, and 30.81%, respectively. Structural alignment between ecdPRK3 and ecdSERKs resulted in a RMSD of 1.92 Å, 1.89 Å, and 1.96 Å for BAK1, SERK1, and SERK2 respectively (Fig. 3). The LRR capping domain and five LRRs of PRK3 aligned well with that of the SERK structures. However, the remainder of the C-terminal portion of the structures are less conserved. The PRK3 ectodomain contains six LRRs whereas SERK family members have five LRRs in their ectodomain. The presence of an extra leucine rich repeat in the PRK3 ectodomain may be due to the difference in function and protein/ligand perception.

The SERK family members all contain two tandemly repeated proline rich regions in the C-terminal end of their ectodomain. The proline rich part, which is known as the Ser-Pro-Pro (SPP) motif, lies in between the LRR and transmembrane region and is a unique feature of the SERK family members^60^. These SPP motifs present in the SERK family have been suggested to act as a hinge, which provides flexibility to the extracellular structure. It has also been suggested that the SPP region is used to mediate interactions with the cell wall^66^. Although ecdPRK3 shares significant sequence and structural similarity with the ectodomains of the SERKs, no proline rich SPP motif is present in the AtPRK3 ectodomain.

The LT/SGxIP motif is very common in plant specific LRR receptors^19^. Along with the SERK family members, this particular motif is also abundantly present in the AtPRK3 ectodomain. The LRR conserved sequence for AtPRK3 is plant specific and is as follows, LxxLxxLxLxxNxLSGxIPxx; this is different from the canonical animal conserved sequence, which is LxxLxxLxLxxNxLxxLpxxoFxx. The plant specific conserved sequence in LRR receptor ectodomains is responsible for their conformation^59,61,67^.

### PRK3 contains a conserved surface patch that is similar to the receptor binding interface on the SERK family of co-receptors

**Figure 4:**
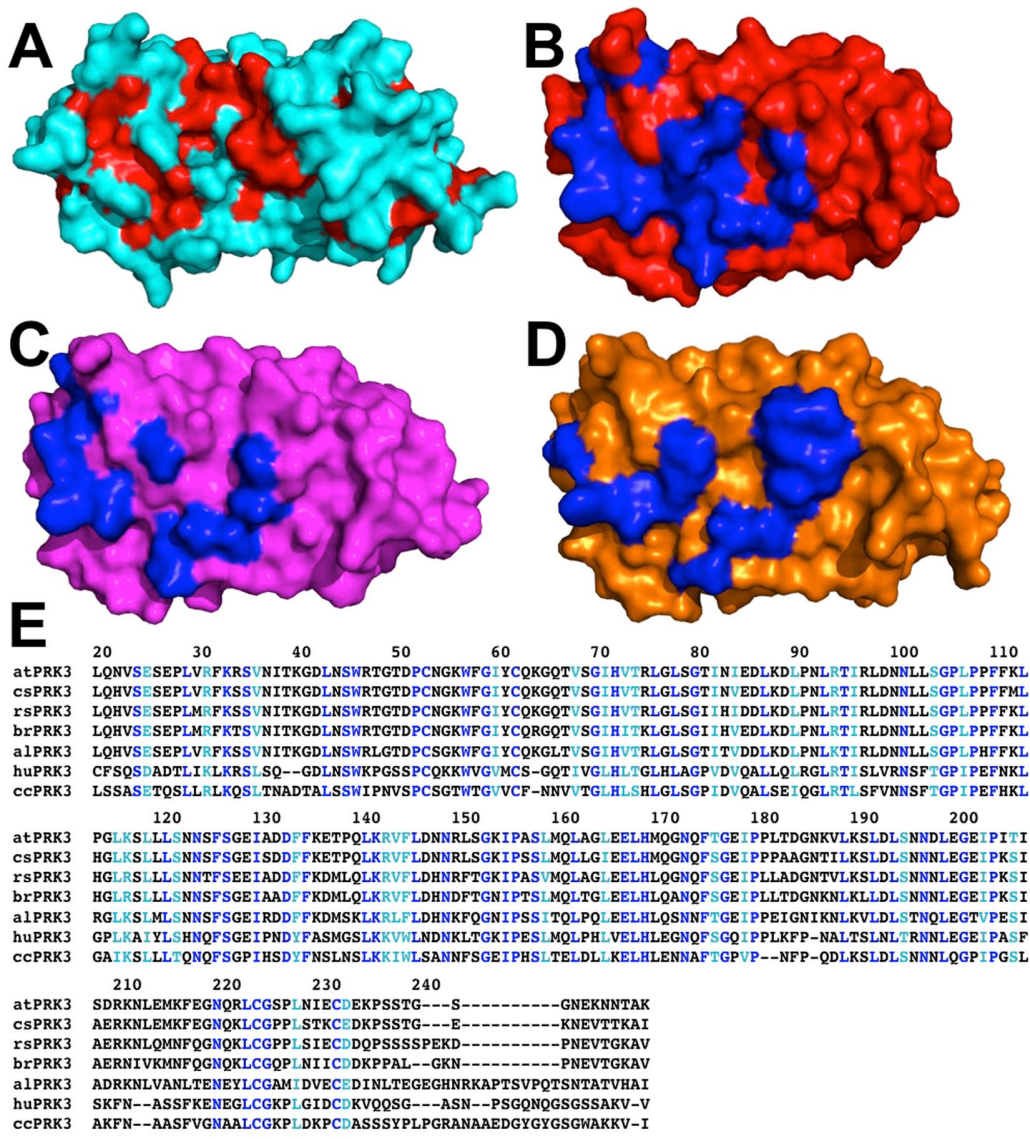
AtPRK3 contains a conserved surface patch that resembles the conserved receptor-binding surface of the SERK family of plant co-receptors. (**A**) The conserved residues that are either identical or similar in all seven selected PRK3 orthologues (in panel **E**) are colored in red on the molecular surface of PRK3 structure, which is depicted in cyan. (**B**) The interface residues of BAK1 that mediate its interaction with BRI1 (pdb id: 4m7e) are colored in blue on the molecular surface of BAK1, which is shown in red. (**C**) The interface residues of SERK1 that mediate its interaction with BRI1 (pdb id: 4lsx) are colored in blue on the molecular surface of SERK1, which is shown in magenta. (**D**) The interface residues of SERK2 that mediate its interaction with PXY (pdb id: 5gqr) are colored in blue on the molecular surface of SERK2, which is shown in orange. (**E**) The amino acid sequences of the extracellular domains of the seven selected PRK3 orthologs are aligned. at, cs, rs, br, al, hu, cc, stand for *Arabidopsis thaliana*, *Camelina sativa*, *Raphanus sativus, Brassica rapa, Arabidopsis lyrata, Herrania umbratica*, and *Cajanus cajan*, respectively. The overall sequence identity between the extracellular domain of *Arabidopsis thaliana* PRK3 and that of the PRK3 of *Camelina sativa*, *Raphanus sativus, Brassica rapa, Arabidopsis lyrata, Herrania umbratica*, and *Cajanus cajan* is 85%, 81%, 77%, 68%, 48%, and 41%, respectively. The residue numbers of *A. thaliana* PRK3 are indicated on the top the sequences. The residues that are identical in all seven orthologues are colored in blue. The residues that are similar in all seven sequences are colored in cyan. Similar residues are defined as: (1) negatively charged side chains as D and E; (2) positively charged side chains as R and K; (3) aliphatic side chains as L, I, and V; (4) aromatic side chains as F, Y, and W; (5) side chains with hydroxyl group as S and T; (6) amide side chains as Q and N. The definition of similar residues is adapted from the BLOSUM matrix.

The SERK family of plant LRR-RLKs are known to function as co-receptors for other LRR-RLKs to mediate hormones and immune responses during plant growth and development^62,64,65^. The crystal structures of several of the extracellular domains of SERK-receptor-ligand complexes are available^62–65,68^. We have examined the binding interfaces between the SERK co-receptors and their LRR receptors. All three SERK members use a conserved surface that is located on the LRR capping domain and the N-terminal portion of the concaved surface of the LRR domain to interact with their receptors to facilitate ligand binding and subsequent signaling (Fig. 4).

We have analyzed the sequence conservation in the extracellular domain of seven PRK3 orthologues, which consist of *Arabidopsis thaliana*, *Camelina sativa*, *Raphanus sativus, Brassica rapa, Arabidopsis lyrata, Herrania umbratica*, and *Cajanus cajan*. (Fig. 4E). The highly conserved residues are mapped on the surface of the PRK3 structure (Fig. 4A). Interestingly, the conserved receptor binding interface in the SERK structures coincides with the conserved surface of the PRK3 structure. Based on the binding surface and ectodomain sequence analysis, we propose that similar to the SERK family members, the AtPRK3 ectodomain concave surface can bind with other LRR receptors or ligands.

### Comparison of ectodomains between PRK3 and other PRK family members

**Figure 5:**
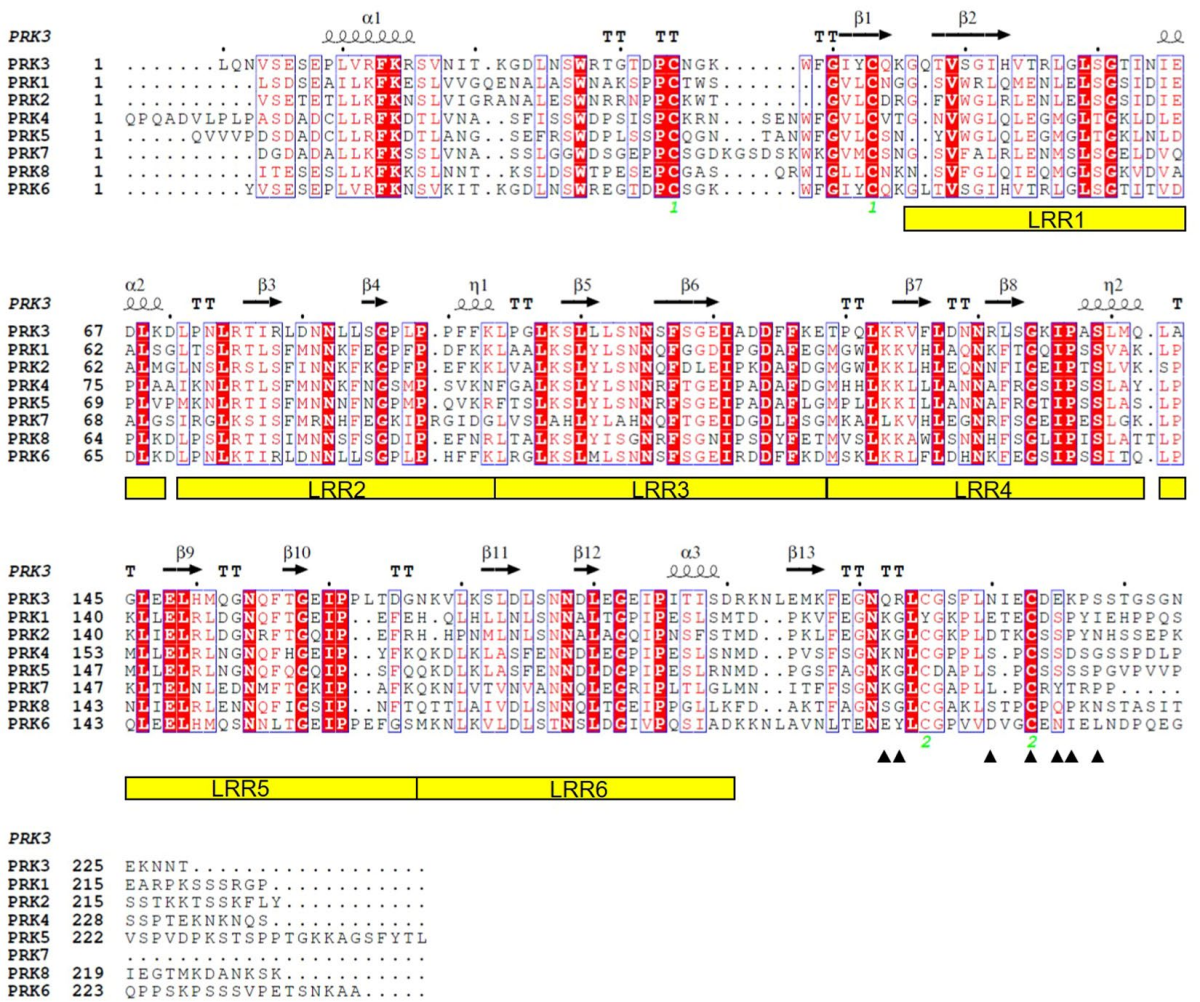
Sequence alignment of the extracellular domains of *Arabidopsis thaliana* PRK1-8. The amino acid sequences of the extracellular domains of *A. thaliana* PRK1-8 are aligned. Each LRR repeat of PRK3 is indicated at the bottom of the sequences. The secondary structural elements of PRK3 are shown on the top with α as α-helix, β as β-strand, η as 3_10_ helix, and TT and turn. The conserved residues are colored in red. The two pairs of cysteine residues that form disulfide bonds in PRK3 structure are indicated in green numerical below the sequence. The AtLURE1.2 interacting residues of PRK6 are indicated with the black solid triangles underneath the sequence.

So far in Arabidopsis eight pollen receptor kinases have been identified. They have been named in numerical order, PRK1, 2, 3, 4, 5, 6, 7, and PRK8^22,69^. It has been suggested that these PRK family members play a significant role in pollen tube guidance during development, ovule targeting, and plant reproduction^37^. We have analyzed the sequence identity of the ectodomains of these receptor proteins, and we have used the crystal structure of ecdPRK3 as a template for homology modeling to evaluate any structural variations present in the other PRK family members. Although the amino acid sequences are highly conserved among all the PRK proteins, which range from 39–70%, certain sequence variability in both the CD and CT can be observed (Fig. 5 and Supplementary Fig. S1). PRK proteins are all predicted to contain six LRR motifs and the crystal structures of both PRK3 and the recently published PRK6 corroborate with this prediction (Fig. 5)^70^. Two conserved cysteine residues present in the N terminal region of both structures form an intramolecular disulfide bond that is responsible for stabilizing the N terminal capping region. Similarly, a pair of conserved cysteine residues is also found in the C terminal region of all the PRK proteins except PRK1 (Fig. 5).

Based on homology modeling analysis with PRK3 ectodomain structure as a template using SWISS-MODEL^71^, all the ectodomains of the other PRKs have a similar C shaped solenoid structure as is present in PRK3 (Supplementary Fig. S1). When aligned with the PRK3 ectodomain structure, the resulting RMSD of PRK1, 2, 4, 5, 6, 7, and 8 are from 0.101 Å, 0.111 Å, 0.077 Å, 0.085 Å, 0.069 Å, 0.181 Å, and 0.078 Å, respectively. The LRR regions and the structural organization are highly conserved among all the PRKs, but certain structural variability can be observed at the CD and CT domains. When compared with the structure of PRK3 ectodomain, we found an elongated loop at the bottom of the concave surface in the CD of PRK1. At the C terminal region, an additional α-helix is present in the case of PRK1. PRK7 contains an extended loop region in the CD. The real implications of these structural variations among different PRK family members are still unknown. More structural and functional studies are required in order to correlate their structures to specific functionality.

A recently published paper on the crystal structure of the PRK6-AtLURE1.2 complex shows some structural differences between PRK3 and PRK6^70^. PRK6 adopts a slightly twisted solenoid shape whereas PRK3 forms a single continuous arc shaped structure. The CT of the ecdPRK6 forms a loop that faces towards the solvent. Previously, it had been reported that the LRR receptors use the lateral surface or the inner concave region to interact with other co-receptors or peptide ligands, but for PRK6, the C terminal loop region interacts with the chemoattractant peptide LURE1.2. The C terminal interacting region of PRK6 mostly contains negatively charged residues that complement the positively charged surface residues of AtLURE1.2, while the same region of PRK3 contains mostly neutral or positively charged residues. Based on sequence alignment of all PRK proteins in Arabidopsis, the AtLURE1.2 interacting residues on PRK6 are variable in other PRKs (Fig. 5). This sequence diversity may explain the differential ligand binding specificity and functionality of PRKs.

## Discussion

The recent structural elucidation of the SERK protein family members has provided useful insights about LRR functionality. Its ligand perception and complex formation has improved our overall understanding of the plant signaling system. It was believed that the SERK proteins only act as co-receptors and do not partake in direct ligand binding^11,59,61^. However, studies on flagellin and BR1 receptors have demonstrated that SERK proteins actively participate in ligand binding and form heterodimers with multiple LRR receptors such as BR1, FLS2, PSKR1, and PXY/TDR^11,59,61,62,64,65^. Our structural comparisons indicate that PRK3 may also function similarly to that of SERK proteins in plant signal transduction cascades.

Numerous studies on LePRKs have indicated that PRK proteins act as signal-transducing receptors by interacting with two other PRK proteins^72^. Multiple LRR-RLKs have been recently identified to engage in the PRK3 mediated responses, such as PRK1, PRK6, PRK8. Genetic and mutational studies on *AtPRK3* along with *AtPRK6, AtPRK8*, and *AtPRK1* have shown defects in pollen tube growth, and *AtPRK3*-*AtPRK1* double mutants have shown impaired responses towards AtLURE1.2. *AtPRK3-AtPRK6* double mutants have also exhibited slow pollen tube growth. Triple mutants specific for *AtPRK3*, *AtPRK6* and *AtPRK8* have shown a reduced fertilization rate than their wild type counterparts^37^.

So far, distinct interactions between AtPRK3 and other pollen receptor kinases have not been determined. It has also been suggested that PRKs interact with several cysteine-rich peptides (CRPs) secreted from pollen and pistil for pollen germination, as well as growth and guidance of the pollen tube, but for *Arabidopsis thaliana* no specific interactions between AtLURE1 and AtPRK3 were observed^21,30,37^. Yang *et al*. also have discovered results demonstrating that AtPRK3 does not interact with the AtLURE1.2^38^. Other pollen receptor kinases such as MDIS1, MIK1, and MIK2 have also been identified in *Arabidopsis thaliana*^38^. It has been shown that MDIS1 and MIK1 interact with LURE1.2, but MIK2 does not^38^. It is still unclear whether any of these AtPRKs, other than PRK6, interact with AtLURE alone, or if there are other unknown CRPs that are specific for these receptors. In addition, it remains to be determined whether the above LRR-RLKs function as pairs in the perception of extracellular ligands. Further *in vitro* and *in vivo* binding assays and functional studies examining the above receptors are necessary in order to identify whether PRK3 pairs with other LRR-RLKs during pollen tube development. Our structural studies of PRK3 have paved the way for future functional investigation of the PRK3 receptor.

## Methods

### Protein expression and purification

To elucidate the structure of AtPRK3, we expressed the extracellular domain of AtPRK3 from *A. thaliana* using baculovirus-mediated insect cell expression. The PRK3 gene encoding residues 20–237 was fused to the secretion signal sequence of hemolin and then cloned into a modified pFastBac1 vector. The secreted protein was first purified by nickel-affinity chromatography using an engineered 6-histidine tag at the carboxyl terminus of the PRK3 protein, and then further purified by size-exclusion chromatography in a buffer containing 20 mM Bis-Tris, pH 6.0, and 100 mM NaCl. The purified protein was concentrated to 5 mg/ml for crystallization. The predicted molecular weight based on the amino acid sequence of the recombinant protein is 25.3 kDa. However, the apparent molecular weight of the purified recombinant protein is approximately 35 kDa presumably due to glycosylation.

### Crystallization and data collection

The recombinant AtPRK3 protein was concentrated to 5 mg/ml. The ectodomain of AtPRK3 protein was subjected to extensive crystallization screening. The protein was crystallized in P4_2_ crystal form using both hanging drop vapor diffusion and sitting drop methods at 18 °C by mixing equal volumes of the purified protein and the crystallization reservoir solution of 0.1 M Tris pH 8.5 and 18% PEG 3350(w/v). For data collection, all crystals were flash frozen in the respective crystallization conditions supplemented with 20% (v/v) glycerol. Diffraction data were collected at the 22-ID (SERCAT) beam line of the Advanced Photon Source (APS). All diffraction data were processed using the HKL2000^73^ suite and their statistics are shown in Table 1.

### Structure determination, refinement and analysis

We have determined the AtPRK3 ectodomain structure by molecular replacement using the SERK1 extracellular domain structure as an initial search model (PDB ID 5IYX). The model of ecd PRK3 structure was built in COOT^74^, and refined with REFMAC5^75^ and PHENIX^76^. The crystals contain two PRK3 molecules in each asymmetric unit cell. The AtPRK3 structure model contains residues 26–233. Two asparagine residues (N37 and N123) in PRK3 are N-glycosylated. One GlcNAc sugar residue on N37 and two on N123 are visible. In addition to the observed N-glycosylation, four cysteine residues are observed to form two disulfide bonds between C53–C62 and C224–C232. The structures were analyzed using the CCP4 suite^77^ and the PISA server^78^, and the figures were made using PyMOL^79^.

### Size-exclusion chromatography

1 mg of purified ecdPRK3 protein was loaded onto a Superdex 200 increase 10/300 GL column (GE Healthcare Life Sciences) in a buffer containing 20 mM Bis-Tris (pH 6.0) and 100 mM NaCl.

### Multiple sequence alignment and homology modelling

Amino acid sequences with single letter code were input to the Clustal Omega online server for multiple sequence alignment (https://www.ebi.ac.uk/Tools/msa/clustalo/)^80^. Clustal Omega uses the HHalign algorithm and its default settings as its core alignment engine. The algorithm is described in Söding, J^81^. The default transition matrix is Gonnet, gap opening penalty is 6 bits, gap extension is 1 bit. Homology modeling analyses of the ECD structures of PRKs with PRK3 ECD structure as a template was conducted with SWISS-MODEL^71^. Structural superposition was rendered in PyMOL^79^.

### Data availability

The atomic coordinates and structure factors have been deposited in the Protein Data Bank under accession code 5WLS.

## Electronic supplementary material

Supplementary Information
