# Supplementary material for: The Extracellular Domain of Pollen Receptor Kinase 3 is structurally similar to the SERK family of co-receptors
Source: Sci Rep. 2018 Feb 12;8:2796. doi: 10.1038/s41598-018-21218-y (PMC5809528; doi:10.1038/s41598-018-21218-y)
Supplement: Supplementary file 1 — Supplementary Information [file 41598_2018_21218_MOESM1_ESM.doc]

**The Extracellular Domain of Pollen Receptor Kinase 3 is structurally similar to the SERK family of co-receptors**

Sayan Chakraborty^1,2^, Haiyun Pan^1,2^, Qingyu Tang^1^, Colin Woolard^1^ and Guozhou Xu^1*^

^1^Department of Molecular and Structural Biochemistry, 128 Polk Hall, North Carolina State University, Raleigh, USA 27695

^2^These two authors contributed equally to this work

*Corresponding Author: Guozhou Xu, gxu3@ncsu.edu

**
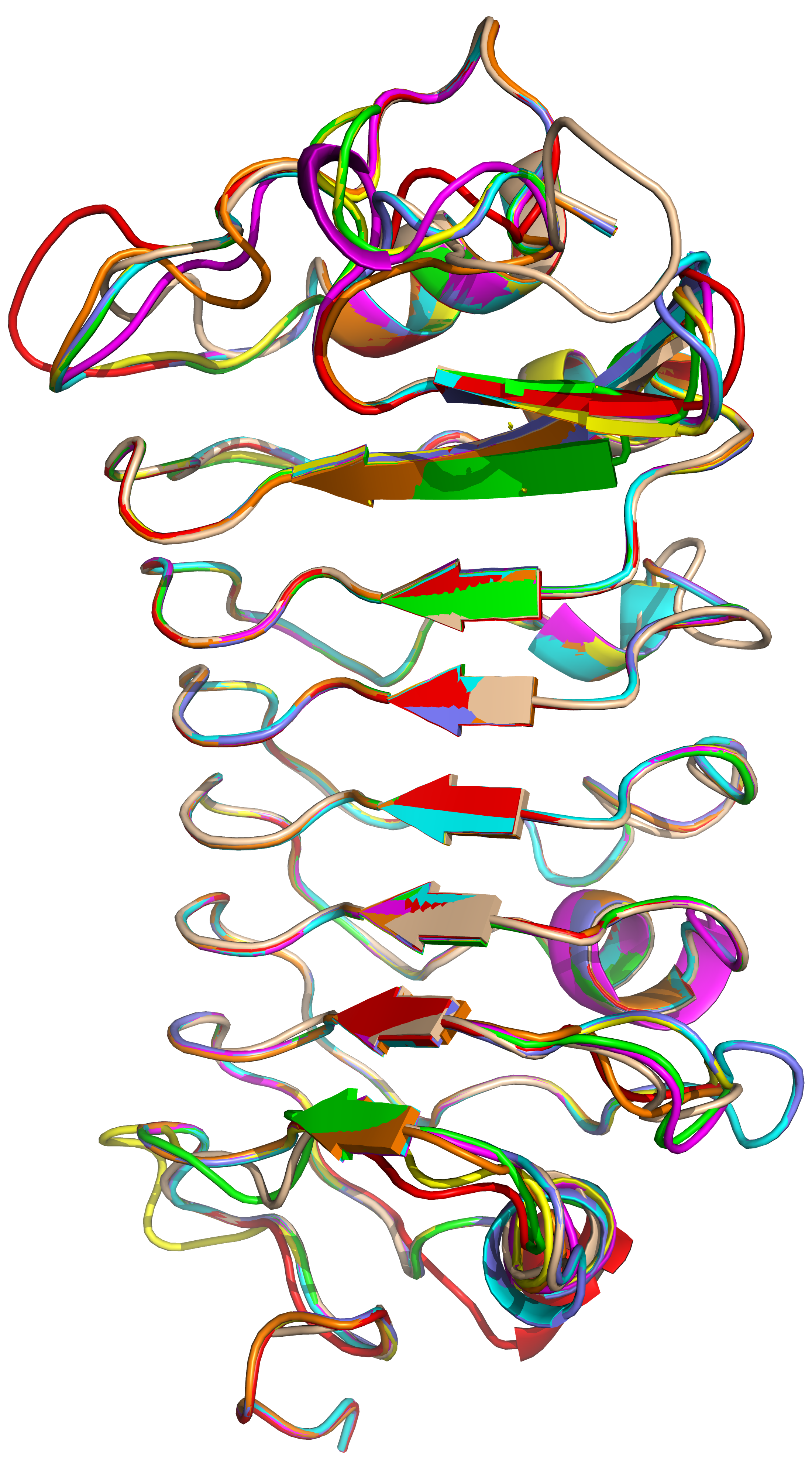
**

**Supplementary Figure S1**: Superposition of the modeled ectodomain structures of *Arabidopsis thaliana* PRK1, 2, 4, 5, 6, 7 and PRK8 with the crystal structure of ecdPRK3. The structures are rendered in PYMOL and depicted here as a cartoon representation. The PRK 1-8 structures are colored in red, orange, cyan, yellow, green, blue, light orange, and magenta respectively. When aligned with the PRK3 ectodomain structure, the resulting RMSD of PRK1, 2, 4, 5, 6, 7, and 8 are from 0.101 Å, 0.111 Å, 0.077 Å, 0.085 Å, 0.069 Å, 0.181 Å, and 0.078 Å respectively.
